# The Cost of Slashing Indirect Costs of Research: A Perspective from the Vantage Point of Scientific Publishing

**DOI:** 10.1002/ntls.70008

**Published:** 2025-05-21

**Authors:** Elaine L. Bearer, Gerard Meijer, Bretislav Friedrich

**Affiliations:** 1 University of New Mexico Health Sciences Center, Albuquerque, New Mexico, USA; 2 Fritz-Haber-Institut der Max-Planck-Gesellschaft, Berlin, Germany

## 1 | Why this Editorial?

Reducing indirect costs of research, which are a key component of research funding, may have the unintended consequence of limiting access to scientific publications, whether for the scientific community or for the public at large. The spread of scientific information and knowledge has been boosted by the 21^st^-century effort to make scientific output openly accessible to everyone. OA publishing has made headlines and been characterized as “one of the ways the world got better in 2023” by *Time* magazine (https://time.com/6550576/13-ways-the-world-got-better-in-2023/?utm_source=pocket_discover0&mc_cid=426239d340&mc_eid=c42af7c76d). The transition to open-access publishing relies on agreements between publishers and research institutions or their consortia, typically represented by research libraries. Given that indirect costs fund libraries that, in turn, compensate publishers for their services to the research community, slashing indirect costs would have detrimental effects on both the spreading of scientific information and the publishers.

In this Editorial, we describe the workings of scientific publishing and its transition to open access, provide historical background on its funding, and spell out the consequences for the dissemination of scientific research results should this funding be cut.

## 2 | The Origins of the Concept of “Indirect Costs”

The notion of “indirect costs” of research was introduced in 1950 by Harry Weaver (1909–1977), the Director of Research from 1946 to 1953 at the National Foundation for Infantile Paralysis (NFIP) (In 1979 the National Foundation for Infantile Paralysis changed its name to the March of Dimes Foundation). The NFIP was founded by Franklin Delano Roosevelt (1882–1945) in 1938 as a charitable organization dedicated to preventing childhood diseases and reducing infant mortality. It spearheaded a national crusade to find a cure for polio, which FDR had contracted in 1921. Distinguished by “his wonderful quality of being bold,” (D. Oshinsky, *Polio. An American Story* (Oxford University Press, 2006), p. 112). Weaver found an antidote for the reluctance of medical schools to accept large extra-institutional research grants, which often surpassed the funds available for all the school’s other activities combined. As Weaver noted, “the acceptance of outside funds has forced the institution [medical school] to expand its physical facilities, its administrative, technical and secretarial staffs, and to spend more money for maintenance and for public utilities ...” (H. Weaver, “A Formula to Determine the Cost of Research,” *Journal of the American Medical Colleges*, July 1950). As described by the historian of medicine, David Oshinsky, “Weaver ran into this problem after only a few weeks on the job. The [NFIP] had [then] agreed to fund a grant proposal from the Bacteriology Department at Harvard, but the university administration had resisted, complaining about the high overhead it would be forced to cover. Weaver responded with a promise to pay a portion of these indirect costs based on a complicated formula he had worked out himself. Harvard then accepted the grant” (D. Oshinsky, *Polio*, p. 113–114). Over time, Weaver simplified his formula for calculating indirect costs (that went to the grantee’s institution) in terms of a percentage of the actual research grant (that went to the grantee). For most governmental grants, the indirect costs to be paid out to the institution were set to 46% of the direct costs made available to the grantee (depending on the institutional policy, the grantees were free to use part of the indirect costs to cover research that was not included in their original research proposal). Other research foundations adopted Weaver’s indirect costs model soon thereafter. Indirect costs and long-term grants revolutionized university research in the U.S. and became a norm. “Harry Weaver turned [research] funding into an art form” (D. Oshinsky, *Polio*, p. 115).

Since Weaver’s time, indirect costs have been instituted by both governmental and not-for-profit research foundations worldwide. Besides expenditures for laboratory facilities, supplies, utilities, administration, and security, indirect costs also include *library costs* (library costs, in turn, typically include journal subscriptions, access to digital books and other digital materials, maintenance of digital catalogs, as well as acquisition of printed materials such as books) and currently typically amount to about 50% of the direct costs.

## 3 | Indirect Costs and the Transition of Academic Publishing to Open Access (OA)

In the olden days, libraries of research institutions paid publishers annual subscription fees for new issues of journals, which served as the principal vehicles for the dissemination of the results of academic research. These subscription fees were *reading fees* that had to be defrayed for the current fiscal year by the end of the previous year. The subscription fees were non-transparent, often hidden from public view by non-disclosure agreements between libraries and publishers. Typically, subscription fees went up at a rate of 3%–4% per year. For large publishers, these fees were bundled (in the so-called Big Deals) in negotiations with the libraries for multiple different journals published by the same press. In the United States and elsewhere, a portion of these subscription costs was reimbursed from the indirect costs, also known as “facilities and administration” (F&A) costs.

Publishers collected subscription fees for their journals to cover their costs of production. When OA publishing came about in the early 2000s, publishers offered individual authors to make their articles freely accessible to everyone to read for an extra fee called an article processing charge (APC). Some funders—such as the National Institutes of Health (NIH) in the United States—even mandated OA publication of the research results obtained under their auspices, which became an important trigger for publishers to create the option of paying APCs in subscription journals. APC costs were not visible to libraries (or research institutions), as they were usually paid out of the direct costs of an individual researcher-author. For authors based at institutions with a subscription to the very journal where they published OA for an APC, this amounted to a double payment for the same publishing service. Such a practice was dubbed “double dipping.”

In order to preclude “double dipping,” the so-called offsetting agreements were instituted between libraries of academic institutions and publishers. The reasoning was that we, the libraries, pay you, the publishers, annually XX Euros in subscription fees, that is, for reading your journal by members of our institution, but you also offer to authors to pay an amount of YY Euros in APCs for their article published OA, that is, free to read for everybody. For that OA article, the libraries would then not have to pay the subscription fee. Rather than asking to lower the subscription fee—and in order to promote OA publishing or, more generally, Open Science (M. Bronner, G. Meijer, V. Yam, and B. Friedrich, “UNESCO Issues a Powerful Endorsement of Open Science,” *Natural Sciences* 2022;2:e10037 https://onlinelibrary.wiley.com/doi/epdf/10.1002/ntls.10037)—offsetting contracts were negotiated that allowed for XX/YY publications from the academic institution with the contract to be published OA at no extra cost (no APC). This fraction XX/YY did not necessarily cover all the articles published by a given publisher and institution with the offsetting agreement in place, as the publishers set the APCs independently from the academic institution.

An alternative to the offsetting agreements came about in the mid-2010s when the first phase of the transformative agreements (TAs) was negotiated and implemented. “A transformative [or transformational] agreement arises when an institution or group of institutions enter into partnership with a publisher to enable a large-scale transition towards open access. A transformational agreement allows the migration of funding from reading towards publishing—whilst recognizing that the subscription element remains an important part of the agreement” (https://www.wiley.com/en-us/network/publishing/research-publishing/open-access/transformational-agreements-at-wiley-how-far-have-we-come).

The annual amount paid to the publisher of a given journal by an academic institution—or a consortium of academic institutions or even a nation-wide consortium such as the DEAL in Germany—was divided by the (average) number of annually published articles by that institution in that journal (in the DEAL-case, the PAR-fee was based on dividing the total amount of money originally paid by all libraries from Germany to a publisher by all the articles with corresponding authors from Germany). This was then taken as a basis for a mutually agreed “Publish-and-Read” (PAR) fee per article, with the acronym APC now standing for Article Publishing Charge. For this PAR fee, each article would be published OA whereas, at the same time, the institution (or consortium) would have reading access to the e-portfolio of the journal. The payment to the publisher thus became article-based. If the number of publications remained the same, the transition from the subscription business model to an OA business model was budget-neutral.

As noted by Max Planck Digital Library’s Ádám Dér, “Dozens of publishers reacted to the growing demand for OA, integrating TAs into their business strategies as they sought to minimize attrition and grow revenue. Tweaking the model to serve their own interests, publishers now sell TAs as a product of their own. As a result, many libraries encounter TAs for the first time as an offer from publishers. It’s no wonder that a significant portion of the library community has come to view TAs as a mechanism designed to entrench the position of for-profit publishers. Such perceptions lead to distrust of those who sign these agreements, who may be seen as complicit in reinforcing the dominance of large publishers” (A. Dér, “What Gets Missed in the Discourse on Transformative Agreements” doi:10.1146/katina-20250212-1).

In the second, ongoing phase of TAs, the thinking has been that what should be paid for are only the costs of publishing and no longer for reading access. At the same time, the costs of publishing should be realistic and transparent and no longer calculated on the basis of the historical subscription rates. Because these payments are due only after an article has been published, the institution has to pay the publisher typically half a year later. This saves, in effect, an equivalent of half of the annual subscription budget. Ádám Dér commented: “[W]ith post-payment models, libraries can guarantee that their financial commitments align directly with the publishing preferences of their authors, allowing them to allocate resources in line with their mission. [...] Institutions and research communities negotiating Tas are making unprecedented levels of new research publicly available, eliminating author-facing APCs and generating substantial savings (Brayman et al., 2024), curtailing the potential of commercial publishers to monetize the works of authors, increasing transparency around the financial streams of scholarly publishing, and optimizing OA publishing workflows with community-driven standards” (A. Dér, “What gets missed...”).

**FIGURE 1 |:**
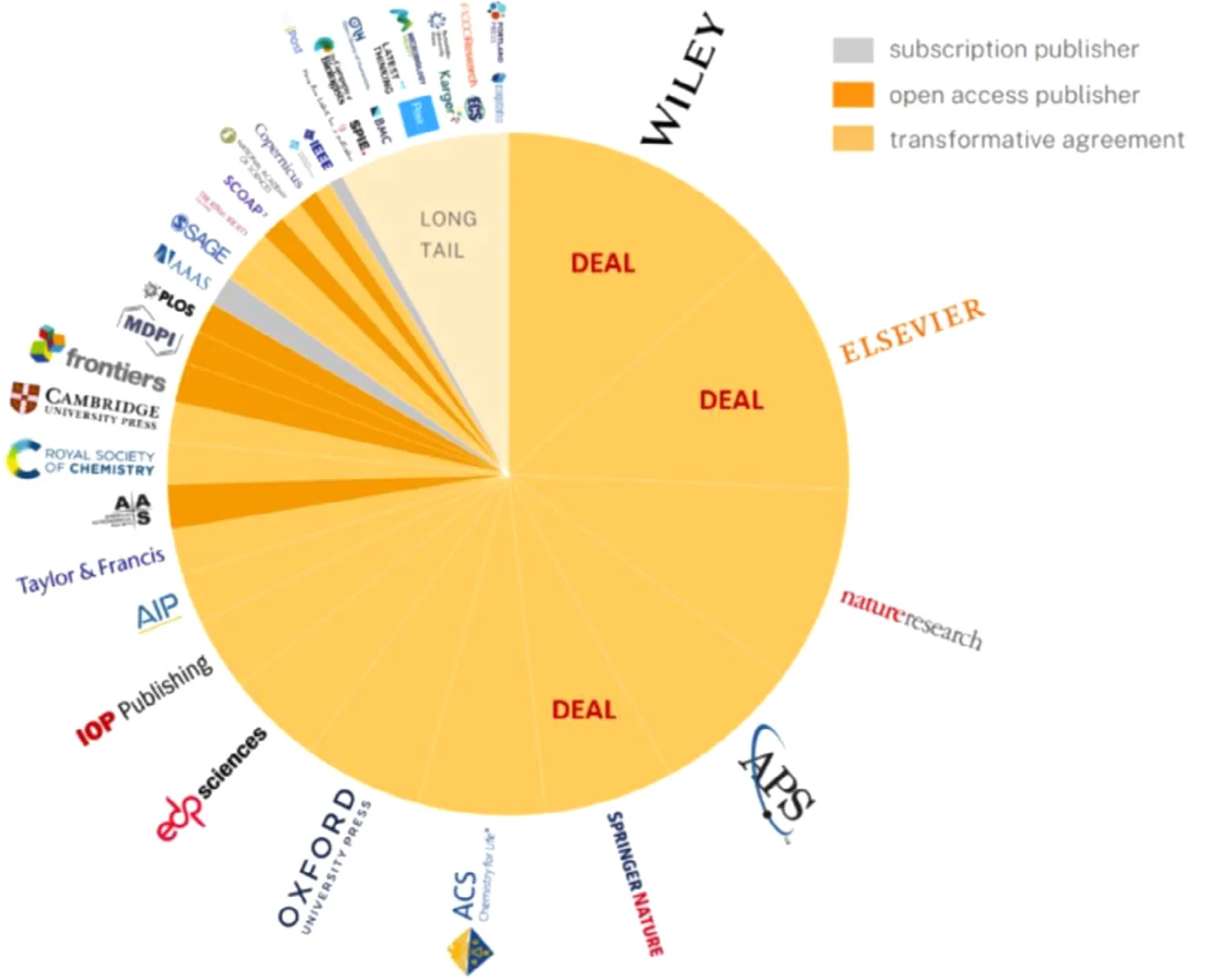
Journal article output of Max Planck Society researchers grouped by publisher. Logos of publishers in the long tail represent existing agreements. *Source:* In-house analysis by Max PlanckDigital Library (MPDL) Big Data Analytics Team, 2025.Adapted fromA.Dér, “What gets missed...”.

Although nation-wide (as exemplified by the DEAL agreements in Germany, see also Figure 1) or consortium-wide (as is the case, e.g., for the University of California system or the Big Ten Universities in the U.S. https://oa2020.org/wp-content/uploads/B16_Session_1_Willmott.pdf), the publishing costs have gone down as a result of the transition to OA, this was not necessarily the case at the institutional level. As publishing scientific results is an integral part of the research enterprise, those institutions that are more research-intensive and, thus, publish more, are likely to pay more in the OA era than institutions that are less research-intensive. Among the latter are also national libraries and other institutions that predominantly read rather than publish. Their costs will decrease compared to what they were in the subscription era. The transition phase provides an opportunity for academic institutions to make the necessary adjustments: Instead of a *Library Budget*, institutions now have to set up an *Information Budget*, out of which all publication costs accrued by their researchers are to be paid. This Information Budget will often be covered by the indirect costs. Having to secure a sufficiently large Information Budget may appear at first as a burden to the leadership of academic institutions. However, in the long run, a plentiful Information Budget proves to be a valuable asset that can and will be used by academic institutions to help attract the best scientists.

Apart from the cost advantages of the OA business model and its fiscal transparency, the copyright for the articles published is not transferred to the publisher (as was invariably the case for the subscription business model) but stays with the author (under a CC-BY attribution license [CC BY stands for Creative Commons, with “by” emphasizing a mandatory attribution https://en.wikipedia.org/wiki/Creative_Commons_license], which stands out as the best choice for maximizing the impact and reach of scientific output (https://deal-konsortium.de/en/)).

## 4 | Impact of slashing “indirect costs” on academic publishing

In response to the release on February 7, 2025 of the new policy guidance by the NIH, Harvard President Alan Garber issued a memorandum “Indirect Costs and Their Impact on Our Research Mission” in which he noted: (A. Garber, “Indirect Costs and Their Impact on Our Research Mission, Harvard University,” Office of the President, 9 February 2025 https://www.harvard.edu/president/news/2025/indirect-costs-and-their-impact-on-our-research-mission/) “The strong, uninterrupted partnership among the federal government, research universities, and industry underpins America’s leadership in biomedical discovery and its application to human health. Federal funding for scientific research has helped make the United States a magnet for outstanding talent, a springboard for ambitious ideas, and a wellspring of rapid and accelerating progress, manifested in an ever-growing list of lifesaving treatments for heart disease, cancer, and genetic diseases, along with technological innovations that have strengthened our economy. It is no wonder that the American model—refined and improved over nearly eighty years—is the envy of the world. [...] [The new policy guidance] will weaken that position by deeply cutting an important but frequently misunderstood source of research funding [indirect costs] in existing and emerging areas of promise. These circumstances are deeply concerning to many of us. [...] Implementing a 15 percent cap on indirect support [...] would slash funding and cut research activity at Harvard and nearly every research university in our nation” (For comparison, the German Wissenschaftsrat in its 2023 Position Statement, see https://www.wissenschaftsrat.de/download/2023/1012-23, recommended to adjust the indirect costs allocated by government grants to meet the needs of the research projects funded).

The slashing of indirect costs would also adversely affect the funding of academic libraries and, with it, the dissemination of the results of academic research (J. Brainard, “DOGE order leads to journal cancellations by U.S. agricultural library,” Science 2025, doi:10.1126/science.zm61f3x). In particular, it would tie the hands of those who negotiate cost-effective TAs with publishers on behalf of the research community. As a result, research works may no longer be available for everyone to read and be relegated to stockpiling behind paywalls. Moreover, should academic libraries go bankrupt, so would academic publishers. Thus, the cost of slashing indirect costs would be to make the scientific output largely invisible.

## Data Availability

All data can be found in the references.

